# Bipolar Membranes Containing Iron-Based Catalysts for Efficient Water-Splitting Electrodialysis

**DOI:** 10.3390/membranes12121201

**Published:** 2022-11-28

**Authors:** Hyeon-Bee Song, Moon-Sung Kang

**Affiliations:** Department of Green Chemical Engineering, College of Engineering, Sangmyung University, Cheonan 31066, Republic of Korea

**Keywords:** water-splitting electrodialysis, bipolar membranes, iron metal compounds, bipolar junction, Fe(OH)_2_EDTA, pore-filling method, complex formation

## Abstract

Water-splitting electrodialysis (WSED) process using bipolar membranes (BPMs) is attracting attention as an eco-friendly and efficient electro-membrane process that can produce acids and bases from salt solutions. BPMs are a key component of the WSED process and should satisfy the requirements of high water-splitting capability, physicochemical stability, low membrane cost, etc. The water-splitting performance of BPMs can be determined by the catalytic materials introduced at the bipolar junction. Therefore, in this study, several kinds of iron metal compounds (i.e., Fe(OH)_3_, Fe(OH)_3_@Fe_3_O_4_, Fe(OH)_2_EDTA, and Fe_3_O_4_@ZIF-8) were prepared and the catalytic activities for water-splitting reactions in BPMs were systematically analyzed. In addition, the pore-filling method was applied to fabricate low-cost/high-performance BPMs, and the 50 μm-thick BPMs prepared on the basis of PE porous support showed several times superior toughness compared to Fumatech FBM membrane. Through various electrochemical analyses, it was proven that Fe(OH)_2_EDTA has the highest catalytic activity for water-splitting reactions and the best physical and electrochemical stabilities among the considered metal compounds. This is the result of stable complex formation between Fe and EDTA ligand, increase in hydrophilicity, and catalytic water-splitting reactions by weak acid and base groups included in EDTA as well as iron hydroxide. It was also confirmed that the hydrophilicity of the catalyst materials introduced to the bipolar junction plays a critical role in the water-splitting reactions of BPM.

## 1. Introduction

A bipolar membrane (BPM) is a unique type of ion-exchange membrane (IEM) in which the anion-exchange layer (AEL) and cation-exchange layer (CEL) are combined. It can decompose water molecules into H^+^ and OH^−^ ions by a strong electric field [1,2]. Traditionally, BPMs have been widely used in water-splitting electrodialysis (WSED) to produce acids and bases from salt solutions [3,4,5]. Recently, BPM has also been utilized in various ion separation systems including continuous electrodeionization [6] and electrochemical energy conversion processes such as fuel cells [7,8], photo-electrochemical cells [9], redox flow batteries [10,11], water electrolysis [12], and acid-base junction flow batteries [13]. Since the performance of these electro-membrane processes is mainly determined by the characteristics of BPM, it is important to clearly understand the water-splitting mechanism and to improve the water-splitting performance of BPMs based on this.

The water-splitting reactions of BPM occur at the bipolar junction where AEL and CEL are in contact, and when CEL is directed to the cathode (−) and AEL is directed to the anode (+) (i.e., a reverse bias condition). When a potential difference, which is a driving force, is applied under the reverse bias condition, the anions and cations present in the BPM move to the external solution through the AEL and CEL, respectively. At this time, the water-splitting reactions are accelerated by a strong electric field (>10^8^ V/m) formed at the bipolar junction. Strathmann et al. reported that the water-splitting reaction rate at the bipolar junction could be accelerated by about 5 × 10^7^ times compared to the solution phase by this strong electric field [2]. In addition, as shown in Equations (1) and (2), a specific functional group (here, B = neutral base) present in the bipolar junction can react with water molecules under a strong electric field for reversible protonation and deprotonation reactions. Therefore, it can consequently accelerate the water-splitting reaction at the bipolar junction [2].

(1) $$B+H_{2}O↔BH^{+}+OH^{-}$$

(2) $$BH^{+}+H_{2}O↔B+H_{3}O^{+}$$

A specific functional group or substance that accelerates the water-splitting reaction as described above can be defined as a water-splitting catalyst, and it includes weakly acidic and weakly basic functional groups, carbon-based graphene oxide derivatives, and transition metal hydroxides and oxides [14,15]. Mel’nikov et al. conducted a study comparing the activities of various water-splitting catalysts and reported that the catalytic activities of Fe(III) and Cr(III) hydroxides were the highest among the materials considered for the study [16]. Table 1 summarizes the activities of various water-splitting catalysts in ascending order [1,16].

In particular, iron, one of the transition metals, has the advantage of being the most abundant on earth, inexpensive, and having little toxicity problem. Therefore, iron compounds have attracted attention as promising water-splitting catalysts, and recently, research results for developing iron-based water-splitting catalysts with improved durability and stability have been reported. For example, Cheng et al. introduced a complex of KFe[Fe(CN)_6_] as a catalyst for water-splitting of BPM into a bipolar junction and fabricated BPMs by a hot-press method. As a result, it was confirmed that the BPM introduced with the KFe[Fe(CN)_6_] catalyst showed superior water-splitting performance than the commercial membrane and could reduce the water-splitting voltage by 43.3% compared to the BPM without the catalyst [17]. Shehzad et al. grew conductive polyaniline (PANI) layer on the surface of Nafion-CEL and uniformly fixed Fe^+3^O(OH) catalyst thereon. At this time, the PANI layer was introduced to prevent the leakage of metal ions and to have stable water-splitting performance of BPM. The prepared BPM showed a low water-splitting voltage (0.8 V), excellent long-term stability, and water-splitting performance [18]. In addition, Wang et al. developed a Fe-MIL-101-NH_2_ catalyst with a metal-organic framework (MOF) structure. It was reported that the optimal loading amount of 0.1 g/L was determined through the current-voltage curve and water-splitting performance evaluation, and the improved water-splitting effect was proven to be affected by the porous structure of the MOF catalyst as well as amino groups and iron ions contained in the catalyst [19]. Ge et al. fabricated thin metal-polymer coordination complex junction-based BPMs. In their study, a catalyst (Fe(III)@PEI) in which Fe and PEI are coordinated through an amine-iron interaction was developed, and excellent water-splitting performance and improved electrochemical stability of the BPMs were confirmed [14]. However, except for the results of Shehzad et al., the water-splitting voltages of the prepared BPMs including the iron-based catalysts were significantly higher than that of the commercial BPMs. Therefore, we believed that the water-splitting performance of the BPMs with iron-based catalysts needs to be further enhanced, and the physical and chemical stability of the catalysts should also be improved.

Meanwhile, for successful use in electro-membrane processes, BPM must be chemically and physically stable in strong acid and base solutions, with low electrical resistance, fast water-splitting rate, and high permselectivity of the ion-exchange layers [20]. In addition, it is very important to develop a low-cost BPM, and for this, it is necessary to consider the BPM fabrication method and materials that can lower the membrane manufacturing cost.

In this work, high-performance/low-cost BPMs including iron-based catalysts for efficient WSED process were developed. In particular, it was attempted to derive the optimal design factors of the iron-based catalysts with excellent performance by comparatively analyzing the water-splitting characteristics of various iron compounds. It was also attempted to improve the stability of the conventional iron hydroxide through a method of forming a complex with inorganic metal nanoparticles or organic ligands. Iron oxide (Fe_3_O_4_) was selected as the inorganic metal nanoparticles, and a porous MOF, zeolitic imidazolate framework (ZIF), was also considered as a catalyst and carrier material. In addition, ethylene-diamine-tetraacetic acid (EDTA), a famous chelating agent, was chosen as the organic ligand compound. EDTA can form a stable complex with transition metal ions and also has weakly basic and weakly acidic functional groups, so it was expected to accelerate the water-splitting reactions at the bipolar junction of BPM. Moreover, a simple method using inexpensive materials was employed for the fabrication of low-cost BPM. In particular, by using a pore-filled membrane prepared with a low-cost polyolefin porous support as a base membrane, BPMs having excellent mechanical properties while being thin (ca. 50 μm-thick) could be fabricated. The prepared water-splitting catalysts and BPMs were systematically characterized through various analysis methods and the BPM properties were compared with those of a commercial membrane.

## 2. Materials and Methods

### 2.1. Materials

(Vinylbenzyl)trimethylammonium chloride (VTAC), trimethylolpropane triacrylate (TMPTA), and styrene (Sty) were purchased from Sigma-Aldrich (St. Louis, MO, USA) and used as monomers for the preparation of the pore-filled anion-exchange membrane. Diphenyl(2,4,6-trimethylbenzoly)-phosphine oxide (TPO, Tokyo chemical industry Co., Ltd., Tokyo, Japan) was used as a photo-initiator and dimethylacetamide (DMAc, Sigma-Aldrich, St. Louis, MO, USA) as a solvent. As a porous support, a polyethylene (PE) separator film (Hipore, Asahi Kasei E-materials, Tokyo, Japan) was utilized. The specifications of the porous substrate used in this work are summarized in Table 2. Polyether ether ketone (PEEK, 450 PF, Mw = 39,200, Vitrex, Lancashire, UK) and sulfuric acid (Samchun, Seoul, Korea) were utilized to prepare a cation-exchange polymer for casting. For the preparation of water-splitting catalysts, iron chloride (FeCl_3_), iron(III) chloride hexahydrate (FeCl_3_·6H_2_O), iron(II) chloride tetrahydrate (FeCl_2_·4H_2_O), ethylenediaminetetraacetic acid (EDTA), zinc nitrate hexahydrate (Zn(NO_3_)_2_·6H_2_O), 2-methylimidazolate (MeIm), 2-propanol (IPA), and sodium hydroxide (NaOH) were purchased from Sigma-Aldrich (St. Louis, MO, USA) and used without any purification. As the commercial IEMs for the property comparison and WSED experiment, Fumatech FBM (Bietigheim-Bissingen, Germany) was used for BPM, and CMX and AMX (Astom, Tokyo, Japan) were employed for cation-exchange membrane (CEM) and anion-exchange membrane (AEM), respectively. These commercially available membranes are high-performance grade membranes that are widely used in various electro-membranes processes including WSED.

### 2.2. Preparations of Reinforced AEM and Cation-Exchange Polymer

**Figure 1:**
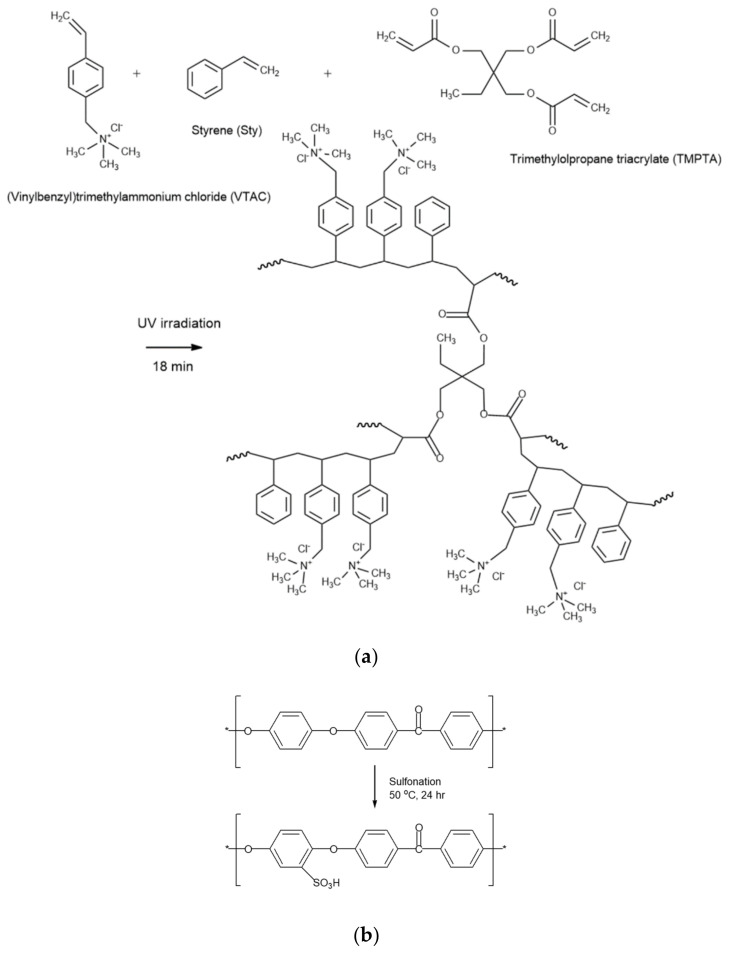
Reaction schemes of (**a**) crosslinked poly(VTAC-Sty) and (**b**) SPEEK.

To prepare the anion-exchange membrane (AEM), VTAC and Sty were mixed at a 1:1 molar ratio, and then 10 wt% of TMPTA as a crosslinking agent and 5 wt% of TPO as a photoinitiator were added to the monomer mixture. After impregnating a PE porous support with the prepared monomer mixture for a certain period of time, and then photopolymerized for 18 min using a UV lamp (TL-K 40W/10R, Philips, Amsterdam, The Netherlands). In addition, to prepare a cation-exchange polymer for casting, 10 wt% of PEEK and 90 wt% of sulfuric acid were added to a four-necked round flask and reacted at 50 °C for 24 h in a nitrogen atmosphere. After the modification reaction was completed, the solution was precipitated in distilled water (DW) and washed with DW several times. The precipitated sulfonated PEEK (SPEEK) was completely dried in a vacuum oven at 80 °C and dissolved in DMAc at 20 wt% to prepare a cation-exchange polymer solution [22]. Figure 1 shows the preparation procedures and chemical structures of crosslinked poly(VTAC-Sty) and SPEEK.

### 2.3. Preparations of Water-Splitting Catalysts

To prepare Fe(OH)_3_, 20 mL of 0.1 M FeCl_3_ was slowly added dropwise to 100 mL of 0.5 M NaOH to cause a precipitation reaction. Thereafter, the precipitated solid was separated by filtration and dried in a vacuum oven at 50 °C for 12 h to obtain Fe(OH)_3_ powder.

To synthesize Fe(OH)_3_@Fe_3_O_4_, an ammonia solution was added dropwise to a solution of 4 mmol FeCl_3_·6H_2_O and 2 mmol FeCl_2_·4H_2_O dissolved in 40 mL of DW to adjust the pH to 11. The resulting black dispersion was stirred at room temperature for 1 h and then refluxed for 1 h to separate the particles and solvent. After that, the separated particles were washed 2–3 times with DW and ethanol, and the resulting particles and 15 mmol FeCl_3_·6H_2_O were sonicated in 10 mL of ethanol for 10 min. The particles were separated from the ethanol solution using a magnet and then dried at 80 °C for 4 h. The dried brown nanoparticles were reacted with 5 mL of ammonia solution while stirring. It was then washed with DW at least 3 times and dried in a vacuum oven at 100 °C for more than 12 h [23].

To prepare Fe(OH)_2_EDTA, 30 mL of a solution prepared by mixing FeCl_3_ and EDTA in a molar ratio of 1:1 was prepared, and then 0.5 M NaOH was added to adjust the pH of the solution to 12. After that, the precipitated solid was separated by filtration, and dried in a vacuum oven at 50 °C for 12 h to obtain Fe(OH)_2_EDTA powder.

To prepare ZIF-8, 3.6 g of zinc nitrate hexahydrate and 8.0 g of 2-methylimidazolate were each dissolved in 200 mL of methanol at 60 °C for 30 min by stirring and then mixed together. After that, the mixture was stirred at 60 °C for 1 h, and 15 mL of this solution was centrifuged at 3000 rpm for 15 min. The separated particles were washed in methanol and centrifuged again at 3000 rpm for 15 min. The obtained particles were dried in a vacuum oven at 50 °C for 12 h, and then further dried at 120 °C for 3 h [24].

To obtain Fe_3_O_4_@ZIF-8, 0.5 g of Fe_3_O_4_, 0.6 g of Zn(NO_3_)_2_·6H_2_O, and 50 mL of DW were mixed and sonicated for 20 min. Meanwhile, 11.5 g of 2-methylimidazolate was dissolved in 5 mL of DW, and the two solutions were mixed and stirred for 10 min. The prepared particles were separated from the solvent and washed 3 times with DW. Finally, Fe_3_O_4_@ZIF-8 particles were obtained by drying in a vacuum oven at 80 °C for 12 h [25].

### 2.4. Fabrication of BPMs

**Figure 2:**
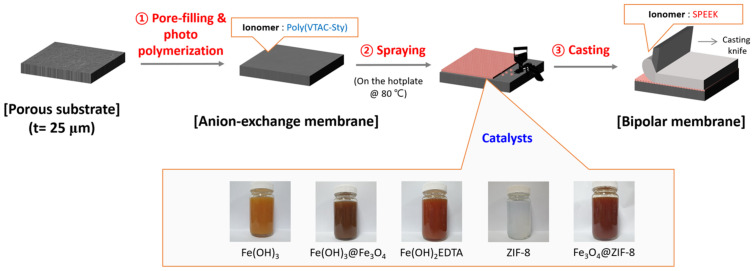
Schematic drawing of BPM fabrication procedures.

After fixing the prepared pore-filled AEM on a glass plate, a catalyst solution in which the prepared metal compounds were dispersed in a dispersion medium of IPA:DW = 1:1 (*v*:*v*) at 0.1 wt% was homogeneously coated on the base membrane surface by a conventional spraying method. After casting the SPEEK solution on the catalyst coated AEM, it was dried in a vacuum oven at 40 °C for 12 h [22]. The BPMs were prepared in size of 5 × 8 cm^2^ with a thickness of about 50 μm and cut to an appropriate size for the following experiments. Moreover, to determine the optimal loading content of catalyst, 0.25, 0.5, 1.0, 1.5, and 2.0 mL of the catalyst solution were sprayed, respectively, to prepare the BPMs. The amount of catalyst used in the fabrication of the BPMs was 0.012, 0.023, 0.046, 0.069, and 0.093 mg/cm^2^, respectively. The BPM fabrication procedures of this study are illustrated in Figure 2.

### 2.5. Characterizations of IEMs and Water-Splitting Catalysts

The electrical resistance (ER) of IEMs was measured in 0.5 M NaCl aqueous solution at room temperature using a lab-made 2-point probe clip cell and LCZ meter. The ER values were calculated using Equation (3) [26].

(3) $$\mathrm{ER}=\left(R_{1}-R_{2}\right)\times A\;\left[\Omega \cdot {\mathrm{cm}}^{2}\right]$$

where *R*_1_ is the resistance of the electrolyte solution and the membrane (Ω), *R*_2_ is the resistance of the electrolyte solution (Ω), and *A* is the effective area of the membrane (cm^2^). Water uptake (WU) was determined by measuring the wet weight (*W_wet_*) and dry weight (*W_dry_*) of the membrane, and was calculated using Equation (4) [27].

(4) $$\mathrm{WU}=\frac{W_{wet}-W_{dry}}{W_{dry}}\times 100\;\left[\%\right]$$

The ion-exchange capacity (IEC) of AEM was determined using the Mohr method. When the IEM reached equilibrium in 0.5 M NaCl, it was washed with DW and then immersed in 0.25 M Na_2_SO_4_ solution for more than 6 h so that Cl^−^ ions in the membrane were completely replaced with SO_4_^2−^ ions. The amount of Cl^−^ in the solution was quantitatively analyzed by titration with 0.01 N AgNO_3_ standard solution. To determine the IEC of cation-exchange membrane (CEM), the membrane was immersed in 0.5 M HCl and, when reached an equilibrium state, washed with DW and then immersed in 0.5 M NaCl for 6 h or more so that Na^+^ ions were replaced with H^+^ of CEM. Thereafter, the concentration of H^+^ ions in the solution was titrated with 0.01 N NaOH solution for quantitative analysis. The IEC values were calculated using the following equation [27].

(5) $$\mathrm{IEC}=\frac{C\cdot V_{s}}{W_{dry}}\;\left[\frac{\mathrm{meq}.}{g_{\mathrm{dry}\mathrm{memb}}}\right]$$

where *C* is the normal concentration of the titration solution (meq./L), vs. is the solution volume (L), and *W_dry_* is the weight of the dried membrane (g). The transport number indicating the selective permeability of IEM was measured by the *emf* method using a 2-compartment diffusion cell, and the values were calculated from the following formula [28].

(6) $$E_{m}=\frac{RT}{F}(2t^{+}-1)\ln\frac{C_{L}}{C_{H}}=\frac{RT}{F}(1-2t^{-})\ln\frac{C_{L}}{C_{H}}$$

Here, *E_m_* is the measured cell potential, *t^+^* is the transport number for CEM, *t^−^* is the transport number for AEM, *R* is the gas constant, *T* is the absolute temperature, *F* is the Faraday constant, and *C_L_* and *C_H_* are the concentrations of NaCl solution (1 mM and 5 mM, respectively). The cell potential was measured by connecting a pair of Ag/AgCl electrodes to a digital multimeter. The tensile strength of IEMs was measured according to international standards (ASTM method D-882-79) using a universal testing machine (34SC-1, Instron, Norwood, MA, USA). The morphological characteristics of the prepared IEMs and catalyst compounds were investigated using a field emission scanning electron microscope (FE-SEM, TESCAN, Brno, Czech Republic). The chemical structures of prepared IEMs and catalyst compounds were also confirmed using Fourier transform infrared spectroscopy (FT-IR, FT/IR-4700, Jasco, Tokyo, Japan). In addition, X-ray diffraction (XRD, miniflex600, Rigaku, Tokyo, Japan) and X-ray photoelectron spectroscopy (XPS, Thermofisher scientific, Waltham, MA, USA) were measured to confirm the structure of the prepared catalyst compounds.

### 2.6. Evaluation of BPM Water-Splitting Capability

The water-splitting performance of BPM was measured using the same 2-compartment cell (membrane effective area = 0.785 cm^2^). First, to measure the water-splitting flux, 150 mL of 0.5 M NaCl was filled in each compartment and Ag/AgCl plate electrodes were placed at both ends. The experiment was conducted at room temperature for 10 min by applying a constant voltage (CV) of 4 V. At this time, the concentration of OH^−^ ions generated over time in the compartment of the AEL was measured using a pH meter, and the cumulative water-splitting flux for a certain period of time was calculated using this value. To determine the water-splitting voltage of BPMs, the 2-compartment cell was filled with each 150 mL of 1 M HCl in the compartment on the CEL and 1 M NaOH in the compartment on the AEL, and then the membrane potential was measured at a constant current (CC) density of 10 A/dm^2^. For the measurement of the membrane potential, a pair of Ag/AgCl reference electrodes were placed close to the membrane, and a current was also applied through the pair of Ag/AgCl plate electrodes to ignore the effect of electrolytic reactions at the electrodes. For the current-voltage (*I-V*) curve measurement, after placing the membrane at the center of the 2-compartment cell, each 140 mL of 0.5 M NaCl was filled in both chambers. A pair of Ag/AgCl reference electrodes were placed near the membrane to measure the membrane potential, and an *I-V* curve was obtained by applying a current at a rate of 0.1 mA/s through a pair of Pt electrodes.

Meanwhile, the chronopotentiometry experiment was performed to evaluate the electrochemical stability of BPMs. For the experiment, each compartment of the 2-compartment cell was filled with 150 mL of 0.25 M Na_2_SO_4_. To measure the membrane potential, a pair of Ag/AgCl reference electrodes were placed near the membrane, and a constant current density of 38.2 mA/cm^2^ was applied for 12 h through a pair of Pt plate electrodes, and the voltage change with time was recorded.

**Figure 3:**
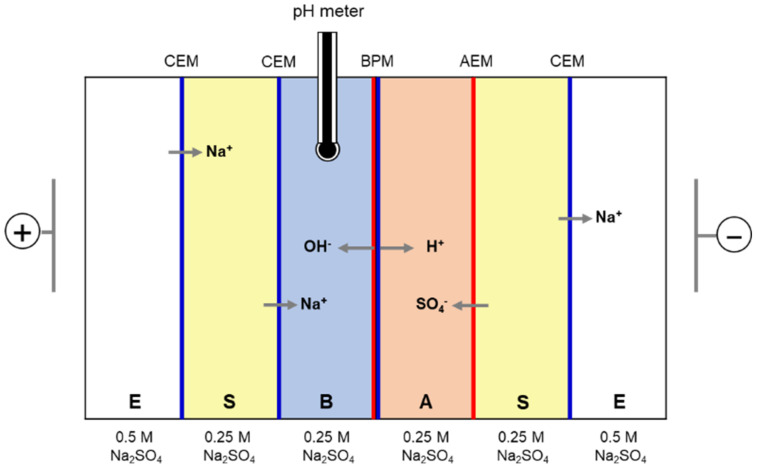
Schematic drawing of 6-compartment WSED cell.

In addition, the WSED experiments were performed using the BPMs prepared with the optimal catalyst content. For the WSED experiments, the lab-made 6-compartment cell was used as shown in Figure 3. In these experiments, CMX and AMX were employed as CEM and AEM, respectively. The effective area of all the membranes and electrodes used in the WSED experiment was 4 cm^2^. A 0.25 M Na_2_SO_4_ and 0.5 M Na_2_SO_4_ were utilized as a salt solution and an electrode rinse solution, respectively, and the solutions were circulated at a flow rate of 50 mL/min using a peristaltic pump. For the WSED experiment, a voltage of 10 V was applied in CV mode by connecting a DC power supply to a pair of Pt plate electrodes. The experiment was carried out for 30 min, and the concentration of OH^−^ ions in the base compartment was measured during the experiment. The water-splitting efficiency was then calculated using Equation (7).

(7) $$\eta =\frac{F\cdot V\left(\frac{\Delta C_{OH-}}{\Delta t}\right)}{I\cdot A}\times 100\left[\%\right]$$

where *F* is the Faraday constant (96,500 C/mol), *V* is the volume of the solution (L), Δ*C* is the change in ion concentration (mol/L), Δ*t* is the change in time, *I* is the current density, and *A* is the effective area (cm^2^).

### 2.7. Stability Evaluation of Catalysts

In order to confirm the stability of the water-splitting catalyst introduced into the bipolar junction, the amount of catalyst eluted to the outside of the BPM at high temperature was measured. BPM samples containing different water-splitting catalysts were prepared in a size of 18 cm^2^, immersed in 100 mL of DW at 60 °C, and the solution was collected every 3 h to measure the concentration of eluted iron ions with a DR4000 spectrophotometer (Hach, Loveland, CO, USA). In addition, the WSED cell experiments were performed before and after the elusion experiment to evaluate the change in the water-splitting performance of the BPMs according to the loss of the catalyst.

## 3. Results and Discussion

**Figure 4:**
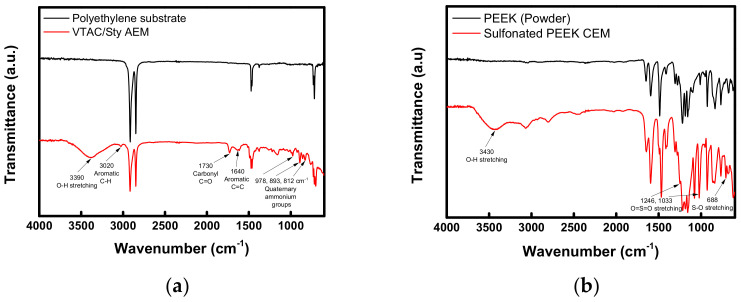
FT-IR spectra of (**a**) PE porous substrate and pore-filled AEM, and (**b**) PEEK and SPEEK CEM.

The FT-IR spectra measured to confirm the chemical structures of AEL and CEL constituting BPM are displayed in Figure 4. As revealed in Figure 4a, the FT-IR spectra of AEM is shown to be significantly different from that of PE support. From the spectrum of AEM, the O-H stretching vibration was confirmed at 3390 cm^−1^, which is due to the hydrophilization of polymer by the introduction of ion-exchange groups [29]. In addition, the C-H aromatic bond was identified from the absorption band at 3020 cm^−1^ [30], and the C=C bond and the aromatic ring were identified from the absorption bands at 1640 and 1390 cm^−1^, respectively [31,32]. Meanwhile, the quaternary ammonium groups were confirmed from the absorption bands of 978, 893 and 812 cm^−1^ [33,34]. In Figure 4b, the FT-IR spectra of PEEK powder and SPEEK are compared together. The O-H stretching vibration indicating the introduction of the ion-exchange groups was confirmed at 3430 cm^−1^ and the O=S=O stretching was also identified from the absorption bands at 1246 and 1033 cm^−1^. Moreover, the S-O stretching of the sulfonic acid groups was confirmed from the absorption band appeared at 688 cm^−1^ [35].

In addition, to confirm the characteristics of AEL and CEL constituting BPM, the monopolar membranes (i.e., AEM and CEM) were prepared and basic analysis experiments were performed, and the results are summarized in Table 3. It was confirmed that the prepared AEM and CEM had a thinner film thickness than that of the commercial membranes (AMX and CMX), and thus showed low ER values, and had almost the same level of ion selectivity and water content compared with the commercial membranes. That is, the prepared AEL and CEL were believed to have the basic properties suitable for fabricating BPM.

**Figure 5:**
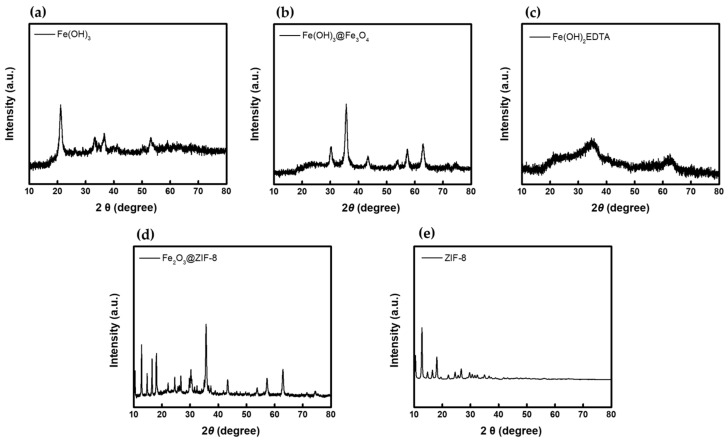
XRD spectra of (**a**) Fe(OH)_3_, (**b**) Fe(OH)_3_@Fe_3_O_4_, (**c**) Fe(OH)_2_EDTA, (**d**) Fe_3_O_4_@ZIF-8, and (**e**) ZIF-8.

The XRD spectra of each compound measured to confirm the structure of the prepared water-splitting catalysts are displayed in Figure 5. The XRD spectrum of Fe(OH)_3_ (Figure 5a) showed major diffraction characteristics at about 35.0°, 42.4°, 53.0°, 57.7° and 63.0°, which is consistent with the reference (JCPDS no. 22-0346) [36]. From the result of Fe(OH)_3_@Fe_3_O_4_ (Figure 5b), the structure of cubic Fe_3_O_4_ (JCPDS no. 86-1354) was confirmed through the peaks appeared at 30.2° (220), 35.4° (311), 43.2° (400), 57.3° (511) and 62.9° (440) [37]. In the spectrum of Fe(OH)_2_EDTA (Figure 5c), however, only broad peaks appeared, which is interpreted as a result of Fe(OH)_2_ forming a complex with EDTA, an organic ligand, resulting in reduced crystallinity. In the graph of Fe_3_O_4_@ZIF-8 (Figure 5d), diffraction peaks assigned to (311), (400), (511), and (440) of Fe_3_O_4_ (JCPDS no. 19-0629) were observed at 35.1°, 42.9°, 56.7° and 62.5°, respectively. Moreover, the structure of Fe_3_O_4_@ZIF-8 was confirmed by observing that additional peaks at 10.3° (002), 12.9° (112) and 18.0° (222) were consistent with those of ZIF-8 (Figure 5e) [38].

**Figure 6:**
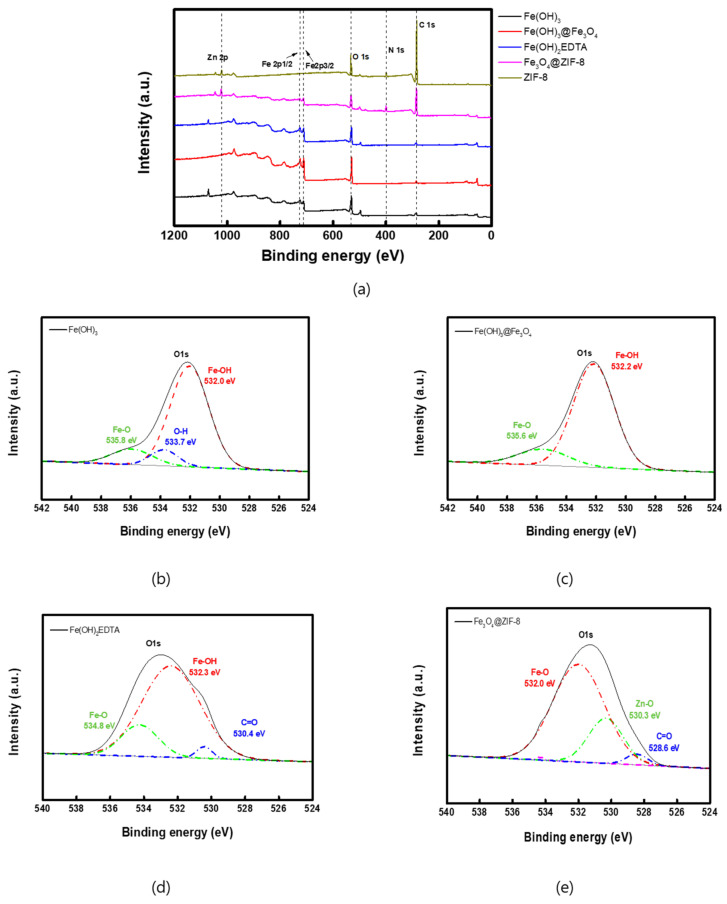
XPS spectra of (**a**) all catalysts, (**b**) Fe(OH)_3_-(O1s), (**c**) Fe(OH)_3_@Fe_3_O_4_-(O1s), (**d**) Fe(OH)_2_EDTA-(O1s), (**e**) Fe_3_O_4_@ZIF-8-(O1s).

The XPS analysis was performed to confirm the structure of the prepared catalysts, and the measured XPS spectra are shown in Figure 6. As revealed in Figure 6a, the peaks for Fe2p1/2 and Fe2p3/2 were observed at 725 and 712 eV, respectively, from the XPS spectra of all catalyst compounds except for ZIF-8 [39]. For more detailed peak analysis, the O1s peak of each catalyst was deconvolved using OriginPro 9.0 software, and the results are shown in Figure 6b–e. Fe-OH peak was observed at 532 eV in the Fe(OH)_3_ spectrum (Figure 6b), and Fe-OH and Fe-O peaks were detected in the Fe_3_O_4_@Fe(OH)_3_ spectrum (Figure 6c) [40]. The bonds of Fe-O and Fe-OH were confirmed at 534.8 eV and 532 eV of the O1s peak of the Fe(OH)_2_EDTA spectrum (Figure 6d), respectively [40]. In the Fe_3_O_4_@ZIF-8 spectrum (Figure 6e), Fe-O bond was found at 532.0 eV, and C=O and Zn-O bonds were confirmed at 530.3 eV and 528.6 eV, respectively [41,42]. These deconvoluted peaks were confirmed to be consistent with the results reported in the references. From the results of the XRD and XPS spectra, therefore, the structures of the catalyst compounds prepared in this study could be confirmed.

**Figure 7:**
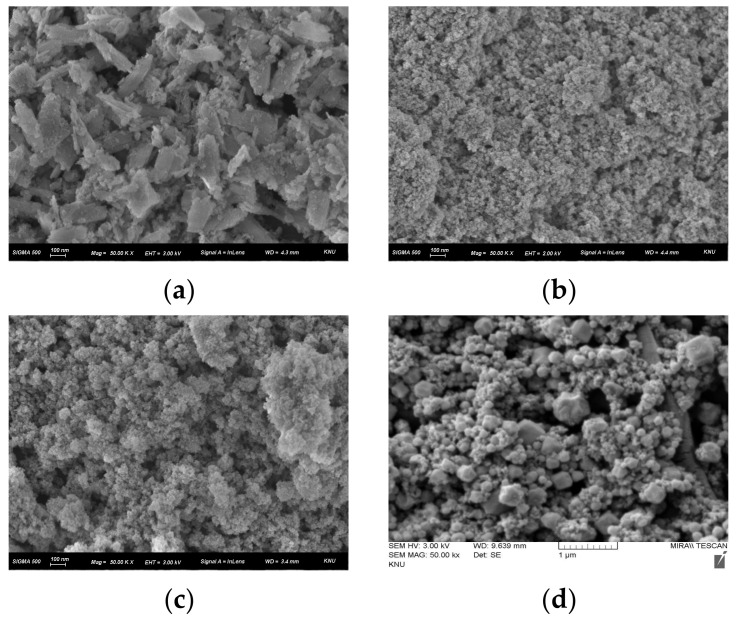
FE-SEM images (50.00k×) of (**a**) Fe(OH)_3_, (**b**) Fe(OH)_3_@Fe_3_O_4_, (**c**) Fe(OH)_2_EDTA, and (**d**) Fe_3_O_4_@ZIF-8.

The FE-SEM images (50.00k×) showing the morphology of the catalyst compounds prepared in this study are displayed in Figure 7. It was confirmed that the catalyst compounds were uniformly coated on the base membrane through spray coating. The image of Fe(OH)_3_ (Figure 7a) showed acicular morphology and the particle size was shown to be 200–300 nm as reported in the literature [43]. In the cases of Fe(OH)_3_@Fe_3_O_4_ (Figure 7b) and Fe(OH)_2_EDTA (Figure 7c), particles having a size of several nm appeared in agglomerated form [23]. Meanwhile, from the image of Fe_3_O_4_@ZIF-8 (Figure 7c), it was confirmed that polyhedral ZIF-8 particles and iron oxide particles were aggregated. As a result, the catalyst compounds prepared in this study were identified as particles having a size of several to hundreds of nanometers, and it was found that they had a large surface area. As the surface area of the catalyst compound becomes larger, the reaction with water molecules increases, so high water-splitting performance could be expected.

**Figure 8:**
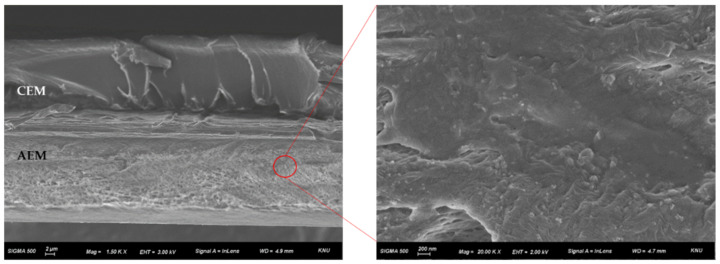
Cross-sectional FE-SEM image (**left**, 1.50k×) of fabricated BPM and higher magnification image (**right**, 20.00k×) of anion-exchange layer.

Figure 8 presents the cross-sectional FE-SEM image (left, 1.50k×) of fabricated BPM and higher magnification image (right, 20.00k×) of AEL. The upper layer of the image is CEL and the lower layer is AEL in the cross-sectional BPM image. Each layer had a thickness in the range of 20–25 μm, and the total thickness (of BPM) was about 50 μm. Unfortunately, however, it was impossible to observe the difference according to the type of catalyst from the cross-sectional image. Meanwhile, the pore-filled structure of the AEL could be observed from the cross-sectional image (left), and it could also be confirmed from the high magnification image (right) that the pores of the PE substrate were completely filled with polymer. It was also revealed that the two ion-exchange layers were well adhered to each other to form a physically stable bipolar junction.

The ER of the BPMs prepared by introducing different water-splitting catalysts was measured. The resistance values of the BPMs were shown to change slightly depending on the loading amount and the kind of catalyst, but there was no significant difference among them, and the average value of the resistance was 0.87 Ω·cm^2^. The resistance of FBM, which is a commercial BPM, was 3.33 Ω·cm^2^ under the same measurement condition. Therefore, it can be seen that the prepared BPM has a significantly lower resistance than that of the commercial BPM, which is mainly due to the reduced thickness. The low ER of the BPM can result in high water-splitting capability and low stack resistance, enhancing the WSED performance.

**Figure 9:**
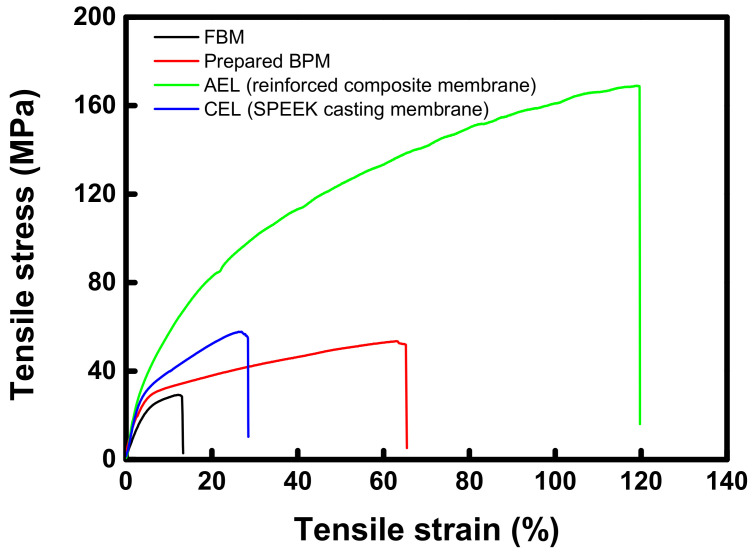
Tensile stress–strain curves of FBM and fabricated membranes (AEM, CEM, and BPM).

Figure 9 shows the tensile stress–strain curves of the FBM and fabricated membranes (AEM, CEM, and BPM). Tensile strength was revealed to be 29.8 MPa for FBM and 52.1 MPa for prepared BPM, and tensile strain was 12.8% for FBM and 65.0% for prepared BPM. Through the results, it was confirmed that the prepared BPM had a toughness several times superior to that of the commercial membrane despite having a film thickness (ca. 50 μm) of much thinner than that of the commercial membrane (ca. 140 μm). This is thought to be because the physical strength of the PE porous support used for the fabrication of the base membrane is excellent [44]. As can be revealed from the results of AEL and CEL shown in Figure 9, the strong mechanical strength of the prepared BPM is mainly due to the AEL used as the base membrane.

**Figure 10:**
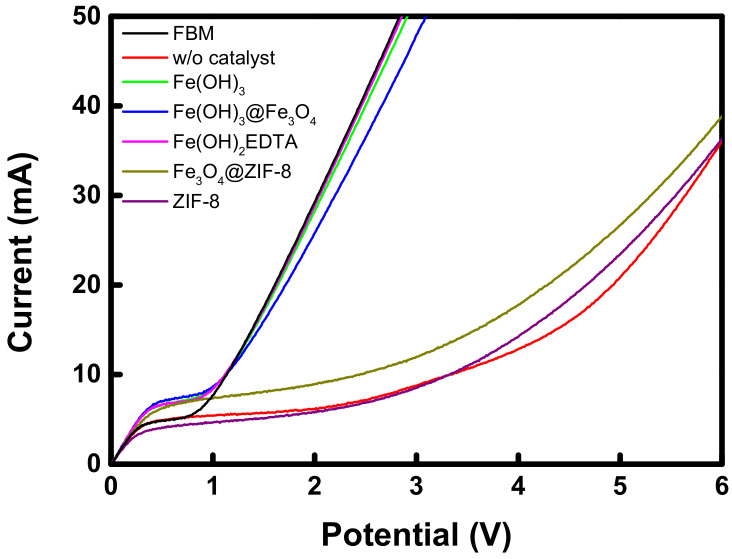
Current-voltage curves of commercial and fabricated BPMs.

The *I-V* curves showing the electrochemical characteristics of the commercial and prepared BPMs are shown by comparison in Figure 10. It can be seen that the prepared BPMs exhibit significantly different *I-V* characteristics depending on the kind of catalyst. In this experiment, the loading amount of each catalyst compound was fixed at 0.01 mg/cm^2^ for comparison. The BPM containing ZIF-8 and Fe_3_O_4_@ZIF-8 showed a very long limiting current density region, similar to the BPM without a catalyst, and a gradual increase in the water-splitting current was observed. This result demonstrates that the catalytic water-splitting effect of ZIF-8 and Fe_3_O_4_@ZIF-8 is insignificant. On the other hand, in the case of the BPMs introducing Fe(OH)_3_, Fe(OH)_3_@Fe_3_O_4_, and Fe(OH)_2_EDTA as catalyst compounds, the short length of the limiting current density region and the steep increase in water-splitting current at about 0.83 V or higher were shown. Table 4 summarizes the water-splitting resistance values of the commercial and prepared BPMs determined through the *I-V* curves. In particular, Fe(OH)_3_ and Fe(OH)_2_EDTA exhibited almost the same level of low water-splitting resistance compared to FBM, which is a commercial BPM.

**Figure 11:**
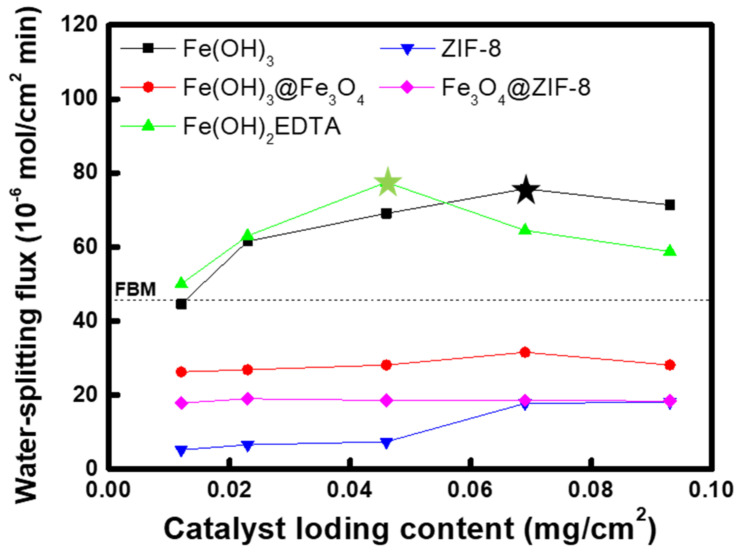
Variation of water-splitting flux of BPMs according to catalyst loading contents.

In order to confirm the water-splitting performance of each catalyst compound in more detail, the water-splitting flux according to the loading amount was measured through 2-compartment cell experiments, and the results are compared in Figure 11. The water-splitting flux of BPMs changed according to the catalyst loading content and the optimal contents were determined to be 0.069 mg/cm^2^ for Fe(OH)_3_ and Fe(OH)_3_@Fe_3_O_4_, 0.046 mg/cm^2^ for Fe(OH)_2_EDTA, 0.093 mg/cm^2^ for ZIF-8, and 0.023 mg/cm^2^ for Fe_3_O_4_@ZIF-8. The tendency of water-splitting flux was almost the same as the results of the *I-V* curves, and the BPMs containing Fe(OH)_3_ and Fe(OH)_2_EDTA of the optimal content were shown to have excellent water-splitting flux superior to that of FBM. In particular, in the case of Fe(OH)_2_EDTA, the optimal loading amount is smaller than that of Fe(OH)_3_, demonstrating that the catalyst activity is relatively higher. On the other hand, Fe(OH)_3_@Fe_3_O_4_, ZIF-8, and Fe_3_O_4_@ZIF-8 showed significantly lower water-splitting flux than FBM. Moreover, the change in the catalyst content does not significantly affect the water-splitting performance, indicating that the catalytic effect of these metal compounds is not that significant.

**Figure 12:**
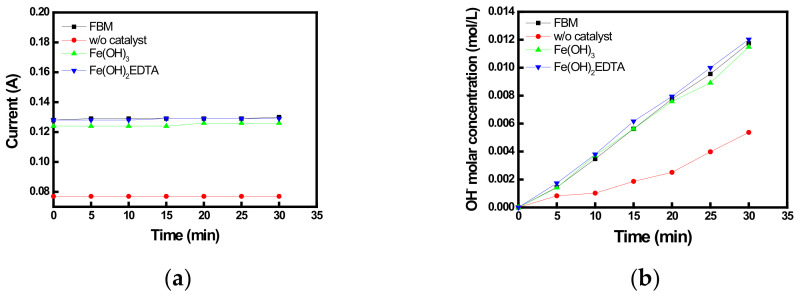
Variations of (**a**) current and (**b**) OH^−^ molar concentrations during the WSED experiments.

In order to compare the BPMs containing Fe(OH)_3_ and Fe(OH)_2_EDTA at the optimum content with the commercial BPM, the WSED experiments using a 6-compartment cell were conducted at CV (10 V) condition, and the results are shown in Figure 12. Similar to the previous results, FBM and BPMs containing Fe(OH)_2_EDTA and Fe(OH)_3_ showed almost equivalent current values and changes in the water-splitting concentration. On the other hand, the BPM without a catalyst resulted in significantly lower current and water-splitting rates compared to those of other BPMs. The water-splitting voltage and efficiency values determined from the WSED experiments are summarized in Table 5. Among the compared membranes, the BPM introduced with Fe(OH)_2_EDTA showed the lowest water-splitting voltage and the highest water-splitting efficiency, indicating the best water-splitting capability. This is thought to be because not only Fe(OH)_3_ but also the carboxylic acid and amine groups contained in EDTA could promote the water-splitting reactions based on the chemical reaction model [14,15].

Meanwhile, ZIF-8, a Zn-based MOF, has been actively applied in various fields because of its simple synthesis method, large surface area, and high chemical and physical stability [45]. From the results, however, it was found that ZIF-8 and Fe_3_O_4_@ZIF-8 did not perform well as water-splitting catalysts in BPMs. To confirm the reason, the contact angles of the base membrane and the catalyst compounds coated on the base membrane were measured, and the results are summarized in Table 6. Among the metal compounds, Fe(OH)_3_, Fe(OH)_3_@Fe_3_O_4_, and Fe(OH)_2_EDTA, which revealed excellent water-splitting performance, exhibited lower contact angles compared to that of the base membrane, which means that the bipolar interface becomes more hydrophilic by the introduced metal compounds. As the hydrophilicity of the bipolar interface of the BPM where the water-splitting reactions occur increases, the supply of water molecules becomes easier and the environment becomes more favorable for the water-splitting reactions. In particular, Fe(OH)_2_EDTA, which had the best catalytic water-splitting performance, showed the lowest contact angle value among the considered metal compounds. On the other hand, ZIF-8 and Fe_3_O_4_@ZIF-8, which exhibited poor water-splitting performance, revealed significantly higher contact angle values than the base membrane, and therefore it is considered that they significantly lowered the hydrophilicity of the bipolar interface. In other words, the water-splitting reactions at the bipolar junction of the BPM were not effectively promoted due to the inherent hydrophobicity of ZIF [46,47].

**Figure 13:**
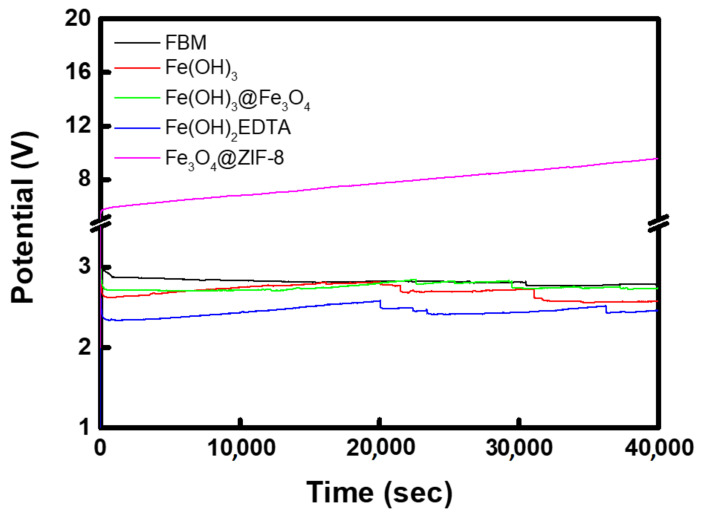
Chronopotentiometry curves exhibiting electrochemical stability of commercial and fabricated BPMs.

To identify the electrochemical stability of the BPMs introduced with metal catalyst compounds, the chronopotentiometry experiment was carried out, and the results are displayed in Figure 13. The BPM introduced with Fe_3_O_4_@ZIF-8 possessed an initial voltage about 2–3 times higher than that of other BPMs, and the water-splitting voltage revealed a tendency to significantly increase over time. This means that the electrochemical stability of the ZIF-8-based catalyst compound is poor, which disturbs the water-splitting reaction at the bipolar junction. In contrast, it was confirmed that FBM and BPMs containing other metal catalyst compounds maintained stable water-splitting voltages at a current density of 38.2 mA/cm^2^ for 12 h.

**Figure 14:**
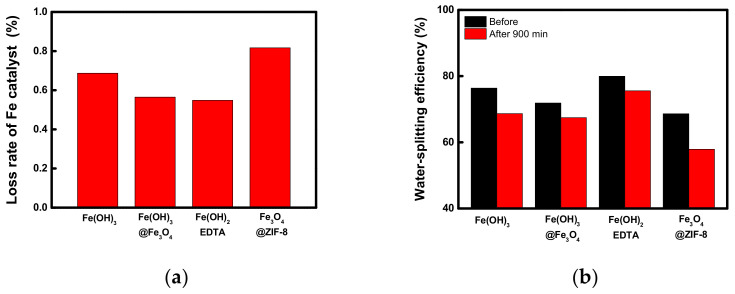
Comparisons of (**a**) loss rate of Fe catalyst and (**b**) changes in water-splitting efficiency of fabricated BPMs containing different catalysts after the elusion test.

Moreover, the elution test was performed at 60 °C for 15 h to confirm the physical stability of the metal catalyst compounds introduced into the BPMs. The catalyst loss rate calculated from the amount of Fe eluted under the above condition is shown for each metal catalyst compound in Figure 14a. As a result, the amount of Fe eluted from Fe_3_O_4_@ZIF-8 was found to be the largest, and the Fe loss rate of Fe(OH)_3_@Fe_3_O_4_ and Fe(OH)_2_EDTA was shown to be lower than that of Fe(OH)_3_. Figure 14b shows the results of comparing the water-splitting efficiency measured through 6-compartment cell experiments before and after the elution test. From the graphs, the water-splitting efficiency of the WSED experiment is shown to decrease after the elusion test, which can be interpreted in relation to the performance change of the BPMs. That is, the decrease in the water-splitting efficiency of WSED became larger as the amount of Fe elution of BPM increased. From the result, the initial water-splitting efficiency and performance maintenance of Fe(OH)_2_EDTA were shown to be the best among the catalyst compounds. This is believed to be because a stable complex was formed by the coordination of Fe to the EDTA ligand as described above, and the weakly acidic and weakly basic groups of EDTA could facilitate the water-splitting reactions at the bipolar junction.

## 4. Conclusions

In this study, several kinds of iron compounds were synthesized and their catalytic effects on the water-splitting performance of BPMs were investigated. The prepared metal catalyst compounds were Fe(OH)_3_, Fe(OH)_3_@Fe_3_O_4_, Fe(OH)_2_EDTA, ZIF-8, and Fe_3_O_4_@ZIF-8 and the structures and morphology of the catalyst compounds were confirmed through various analyses such as FT-IR, XRD, XPS, and FE-SEM. An AEM was prepared by filling crosslinked poly(VTAC-Sty) into a PE porous support, and after spraying a catalyst layer, SPEEK was cast to prepare a BPM with a thickness of about 50 μm. The BPMs prepared with a PE porous support revealed several times superior toughness compared to that of Fumatech FBM owing to the excellent mechanical strength of the reinforcing material. In addition, as a result of various electrochemical analyses, it was found that the BPMs introduced with Fe(OH)_3_, Fe(OH)_3_@Fe_3_O_4_, and Fe(OH)_2_EDTA exhibited the water-splitting performance almost equivalent to that of FBM. The optimal catalyst loading content was determined to be 0.069 mg/cm^2^ for Fe(OH)_3_ and Fe(OH)_3_@Fe_3_O_4_, 0.046 mg/cm^2^ for Fe(OH)_2_EDTA, 0.093 mg/cm^2^ for ZIF-8, and 0.023 mg/cm^2^ for Fe_3_O_4_@ZIF-8. At the optimal catalyst loading, Fe(OH)_2_EDTA showed the highest catalytic activity for water-splitting reactions among the compared metal compounds, and at the same time showed the most excellent electrochemical and physical stability. This was interpreted because a stable complex was formed through the coordination between Fe and the EDTA ligand, the bipolar junction became hydrophilic by the metal compound, and the weakly acidic and weakly basic groups of EDTA, as well as iron hydroxide, promoted the water-splitting reactions. On the other hand, it was also found that ZIF-8-based metal compounds did not promote the water-splitting reaction at the bipolar interface due to their poor stabilities and relatively high hydrophobicity. When comparing the water-splitting properties of the BPMs embedded with iron-based catalysts developed so far, mentioned in the Introduction, the BPM with Fe(OH)_2_EDTA prepared in this study showed the lowest level of water-splitting voltage among them. From the results of this work, the possibility of the fabrication of low-cost BPMs with superior water-splitting capability compared to commercial membranes was also confirmed.

## Figures and Tables

**Table 1:** Catalytic activity of ionogenic groups [16].

Catalyst	Catalytic Activity of Ionogenic Groups (*k_L_*, s^−1^)
-N(CH_3_)_3_	0
-SO_3_H	3 × 10^–3^
Ni(OH)_2_	5.6 × 10^–3^
-PO_3_H-	3 × 10^–2^
=NH, -NH_2_	10^–1^
≡N	1
-COO^−^	10
-PO_3_^2−^	10^2^
Cu(OH)_2_	2.6 × 10^2^
Fe(OH)_3_	1.1 × 10^3^
Cr(OH)_3_	1.5 × 10^3^

**Table 2:** Specifications of porous substrate used in this work [21].

Parameter	Property
Structure	Single layer
Composition	High density polyethylene
Thickness (µm)	25
Gurley (s)	21
Porosity (%)	40
*T_m_* (°C)	138

**Table 3:** Properties of commercial and prepared AEMs and CEMs.

Membranes		Thickness (μm)	ER (Ω·cm^2^)	TN (−)	IEC (meq./g)	WU (%)
AEM	AMX	135	2.54	0.985	1.56	22.1
	Poly(VTA-Sty)	23	0.92	0.988	2.14	20.8
CEM	CMX	165	2.73	0.978	1.98	33.1
	SPEEK	25	0.72	0.980	1.57	31.1

**Table 4:** Water-splitting resistances determined from the *I-V* curves of commercial and prepared BPMs.

BPM	Water-Splitting Resistance (Ω∙cm^2^)
FBM	33.25
w/o catalyst	536.1
Fe(OH)_3_	34.13
Fe(OH)_3_@Fe_3_O_4_	38.22
Fe(OH)_2_EDTA	33.25
ZIF-8	402.1
Fe_3_O_4_@ZIF-8	458.4

**Table 5:** Water-splitting voltage and efficiency values determined from the WSED experiments.

BPM	Water-Splitting Voltage (V)	Water-Splitting Efficiency (%)
FBM	1.32	78.1
w/o Catalyst	1.90	59.8
Fe(OH)_3_	1.31	78.2
Fe(OH)_2_EDTA	1.22	79.9

**Table 6:** Average contact angle values of base membrane (w/o catalyst) and various catalysts coated on base membrane.

Catalyst	Average Contact Angle (Degree)
w/o catalyst	55.20
Fe(OH)_3_	41.28
Fe(OH)_3_@Fe_3_O_4_	41.25
Fe(OH)_2_EDTA	23.79
Fe_3_O_4_@ZIF-8	72.46
ZIF-8	79.19

## Data Availability

Not applicable.
